# A272 EVIDENCE OF SEX DIFFERENCES IMPACTING PAIN SIGNALING BY LUMINAL MEDIATORS IN IRRITABLE BOWEL SYNDROME

**DOI:** 10.1093/jcag/gwac036.272

**Published:** 2023-03-07

**Authors:** S Ables, A Bennett, S Vanner, A Lomax, D Reed

**Affiliations:** Queen's University, Kingston, Canada

## Abstract

**Background:**

Irritable bowel syndrome (IBS) is more than twice as common in women and female patients report more severe abdominal pain. This suggests that sex-specific mechanisms may contribute to the pathophysiology of IBS. Many IBS patients have altered gut microbiota and luminal meditators, implicating the gut microbiota in abdominal pain. These luminal mediators can alter excitability of visceral nociceptors, thus potentially contributing to abdominal pain in IBS. Furthermore, in a subset of IBS patients a low FODMAP diet (LFD) reduces the effect of luminal mediators on pain sensing neurons. The LFD may improve abdominal pain at greater rates in women, and numerous putative mechanisms contributing to abdominal pain in IBS are susceptible to sex-specific mediators. However, it is unknown whether luminal mediators have similar effects on visceral pain signaling in both males and females. We hypothesize that luminal mediators will cause greater differences in neuronal excitability and pain signaling in female mice due to sex-specific factors.

**Purpose:**

To determine whether FS from IBS patients (IBS FS) affects nociceptors from female mice more than nociceptors from male mice.

**Method:**

Neurons from dorsal root ganglia from male and female mice were incubated overnight in media containing fecal supernatant (FS) from IBS patients (N=2 females) before and after the LFD, or healthy controls (HC, N=1 female and 1 male). Ratiometric Ca^2+^ imaging with FURA-2-AM was employed to quantify TRPV1 channel sensitization following application of capsaicin (100nM for 1 minute) as a measure of neuronal excitability. Data was analyzed using chi-squared test as well as two-way and mixed-effects model ANOVA as appropriate, followed by Sidak’s multiple comparisons test.

**Result(s):**

IBS FS caused a 177% larger Ca^2+^ influx in response to capsaicin compared to HC FS in female mice (p=0.0148, N=6-7 mice, neurons=43-49). In male mice, IBS FS increased Ca^2+^ influx by only 13% compared to HC FS (p=0.79, N=5 mice, neurons=28-35). In female mice, 117% more neurons responded to capsaicin after incubation with IBS FS versus HC FS (p=0.0004), while in male mice, only 17% more neurons responded following incubation with IBS FS (p=0.46). Finally, FS from the same IBS patients following a LFD reduced neuronal Ca^2+^ influx by 39% compared to IBS FS in female mice (p=0.0434, N=4-6 mice, neurons=18-49). In male mice, LFD FS reduced Ca^2+^ influx by 11% versus IBS FS (p=0.98, N=5 mice, neurons=28-35).

**Conclusion(s):**

Nociceptive neurons from female mice are more sensitive to the pro-nociceptive effects of FS from IBS patients, as well as a reduction of these excitatory effects following the LFD. This suggests a potential role of sex hormones in pain signaling in IBS.

**Disclosure of Interest:**

None Declared

